# Household characteristics of persons with complex care needs in the community: A preliminary study

**DOI:** 10.1002/nop2.631

**Published:** 2020-09-23

**Authors:** Kyoko Yoshioka‐Maeda, Hitoshi Fujii

**Affiliations:** ^1^ Department of Health Promotion National Institute of Public Health Saitama Japan; ^2^ Department of Medical Statistics, School of Nursing Mejiro University Saitama Japan

**Keywords:** care need, community, family care, public health nursing

## Abstract

**Aim:**

To identify the characteristics of households of persons with complex care needs and clarify the care period length required to resolve their primary health issues.

**Design:**

A descriptive cross‐sectional study design.

**Methods:**

We collected registered data and nursing records from 155 households living in City A within the Tokyo Metropolis. They were designated as complex care cases by the home care and life support centre and needed a multidisciplinary approach to care from April 2018–July 2019.

**Results:**

Most households comprised an elderly with a single adult child (*N* = 47, 30.3%). Mental illness and economic distress overlapped as the most common issues. The mean length of care period was 147.7 days (*SD* = 120.6). The log‐rank test showed that single persons took significantly less time to resolve their primary health issues than elders with single‐child households or single‐person <65‐year‐old households.

## 1 INTRODUCTION

Health inequity and social determinants of health affect public health (Marmot, 2017). In community settings, the number of primary care persons with complex health issues and social care needs has been increasing, with healthcare professionals having encountered difficulties in supporting them (McGregor et al., 2018). Persons with complex care needs and mental health problems face barriers in the use of healthcare services such as experiencing stigma and social deprivation (Stergiopoulos et al., 2018). Furthermore, socio‐economic status also affects the complexity of their health issues (Webster et al., 2019). In community settings, various departments of the health and welfare system have supported households of persons with complex care needs because their health issues were too complicated to be managed by only one department. Thus, multidisciplinary approaches and seamless healthcare systems are needed to support them (Napoles et al., 2017). However, little is known about the characteristics of households of persons with complex care needs in community settings (Yoshioka‐Maeda, 2019).

## 2 BACKGROUND

Persons with complex care needs may have multiple chronic conditions, mental health problems, and be socially vulnerable (Buja et al., 2018). Their biopsychosocial problems include low health literacy and financial constraints (Hochman & Asch, 2017). To identify the complexity of persons’ health issues, several assessment tools have been developed in Western countries; these tools include several dimensions: health and well‐being, social environments, health literacy, communication, patient–practitioner relationships and social supports (Shukor et al., 2018; Pratt et al., 2015). According to one report, the mean length of stay regarding complex cases was 34.1 days, and their complexity was associated with the relative burden for physicians and nurses in hospital settings (Yoshida et al., 2019). However, little is known about family‐related dimensions of persons with complex care needs in community settings because previous studies have been conducted in hospital settings and have focused only on the patients themselves, not in the context of their families or households. A literature review showed that persons with complex care needs have personal and family‐related health issues, with their families also having health issues (Yoshioka‐Maeda, 2019). Thus, healthcare professionals should consider the factors regarding the family members of persons with complex care needs who are members of their households.

Complex cases are also called ‘‘high‐needs or high‐cost patients’’ due to their complex health issues and socio‐economic status. Furthermore, 20% of such persons make up 80% of all medical expenditures because of aspects of their care such as repetitive hospital admissions and emergency department visits. They also need more intensive primary care in community settings than persons with low health risks (Hochman & Asch, 2017). A previous study showed that home visits and team‐based care for persons with complexities reduced the number of emergency department visits and hospitalizations (Ritchie et al., 2016) and the average length of stay per admission (Shukor et al., 2018). However, these studies were conducted in clinic‐based settings and measuring their outcomes was seen as medical expenditures. Therefore, little is known about how many days are required to resolve the primary health issues of persons with complex care needs living at home (McGregor et al., 2018).

With the populations rapidly ageing and birth rates rapidly declining, Japan faces a declining overall population. To supplement the declining labour force, the national government has created a system of mutual assistance at the regional level to enhance social inclusivity by promoting self‐help within communities (Ministry of Health, Labour, & Welfare, 2017). Thus, since 2006, each municipality has worked with an outsourcing contractor and opened a community‐based integrated care centre for elderly people in their local junior high school district (Ministry of Health, Labour, & Welfare, 2016). However, these systems—focused on individual care—could not cover households, such as households of persons with complex care needs that comprise one elderly person with a single adult child. This is because these systems assume that an adult child living with their parent can support them. However, parents in their 80s who are receiving pensions are often supporting their children in their 50s who have experienced social withdrawal and been unemployed for a long time. As Japan has a culture of shame, the poverty of the children involved may be hidden by their parent's financial strength but is gradually revealed as their parent's care needs increases (Yoshioka‐Maeda, 2020). This situation is so common that it has been named the ‘‘8050 issue’’ (Ministry of Health, Labour, & Welfare, 2019). Thus, identifying the characteristics of households of persons with complex care needs is vital to enable public health nurses (PHNs) to support them in community settings. PHNs have played a central role in supporting community‐dwelling people and identifying community health needs through the care of individuals and their families (Ministry of Health, Labour, & Welfare, 2013). However, to the best of our knowledge, there are no reports on the characteristics of households of persons living in their homes with complex care needs.

### 2.1 Research question

For early detection and adequate provision of care for persons with complex care needs living in their homes, we aimed to reveal the characteristics of households of persons with complex care needs and the length of the care period required to resolve the primary health issues in each household.

## 3 METHODS

### 3.1 Design

A descriptive cross‐sectional study design was used to reveal the characteristics of persons with complex care needs in different households.

### 3.2 Sample definition

Based on previous studies (Buja et al., 2018; McGregor et al., 2018; Yoshioka‐Maeda, 2019), the households of persons with complex care needs living at homes are defined as fulfilling the following criteria: (a) person has multiple issues such as health, welfare, social, legal and economic issues, (b) person has various health and welfare departments supporting them, (c) healthcare professionals supporting them have experienced significant difficulties in assisting them with a daily routine.

### 3.3 Setting and sample

The study sample were persons with complex care needs living in City A who were registered as requiring assistance to maintain their community lives. The population of City A was approximately 570,000 with 320,000 households. It was a suburban area in the Tokyo Metropolis. The proportion of the population considered to be “agedˮ was 22.1%, with 10.3% of the population consisting of children aged 15 years or below.

To support the persons with complex care needs in different households, City A has operated the home care and life support centre since April 2018. Various health and welfare departments have supported persons with complex care needs in different households because their health issues were too complicated to be managed by only one department. The centre has coordinated with the relevant staff members of each department to create a seamless healthcare system.

**FIGURE 1:**
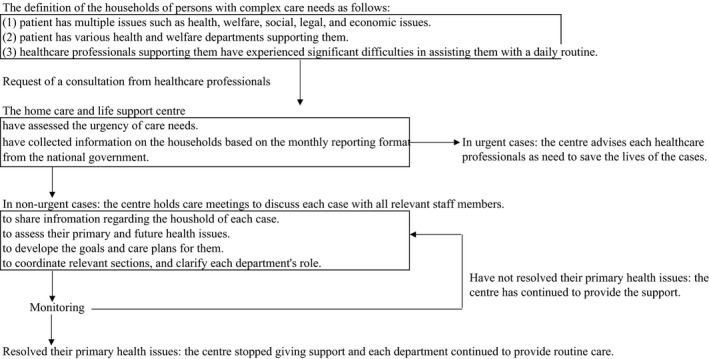
The flow of the consultation for the persons with complex care needs in different households referred to the home care and life support centre in City A

Figure 1 shows the flow of the consultation for the persons with complex care needs in different households referred to the care centre in City A. Healthcare professionals who have been working in the community and assessed that their cases fulfilled the criteria of persons with complex care needs requested support from the centre. The staff of the centre: PHNs, social workers, and psychiatric social workers had assessed the urgency of care needs by using the monthly reporting format from the national government. Additionally, the staff collected information on different households based on this format and analysed their complex care needs and the fundamental causes of primary health issues. The director and department chief were PHNs and had supported the staffs' assessments. In urgent cases, they advised each healthcare professional as needed to save the persons' lives. In non‐urgent cases, the centre held “care meetingsˮ to discuss each case with all relevant staff members. Through these meetings, the staff shared information regarding the households, assessed their primary and future health issues, developed goals and care plans for them, coordinated relevant departments and clarified each department's role. The centre monitored the progress of care and judged whether each department should continue supporting each person with complex care needs. In cases where the primary health issues were resolved, the centre stopped giving support and each department continued to provide routine care.

From April 2018–July 2019, 155 households (227 persons) living in City A were registered as high‐needs cases of the centre. We could not calculate sample size as the number of cases of the centre was limited.

### 3.4 Data collection

We developed a research team with two PHNs, who are the director and the department chief of the centre. The centre has registered high‐needs cases data of each of the households based on the monthly reporting format from the national government for the sake of determining subsidization. These data were entered by the staff in charge of supporting each household. PHNs combined these registered data and their nursing records. The Institutional Review Board approved this study at the institute of the principal researcher; the two PHNs provided anonymized nursing data to the researchers. The data included information as follows:

#### 3.4.1 Demographic data

Demographic data included gender, age and the living place of the households at the end of July 2019.

#### 3.4.2 Type of household

Based on the database obtained from the national government consisting of monthly reports, we used seven categories of households: (1) single‐person under 65‐year‐old household (a single‐person household <65 years old), (2) single‐person over 65‐year‐old household (a single‐person household ≥65 years old), (3) elderly couple household, (4) single‐mother/father household, (5) elderly person with a single‐child household, (6) three‐generation household and (7) other.

#### 3.4.3 First consultant departments

The nursing records identified which department of the care centre was the first consultant for each case.

#### 3.4.4 The length of the care period needed to resolve the primary health issue in each household

We calculated the length of care period needed to resolve the primary health issue in each household from nursing records. The two PHNs identified and collected data regarding whether the primary health issues of the households were resolved or at the end of July 2019. Where there was no need for professional support to resolve the primary health issue of households with complex care needs, PHNs judged the primary health issues as being resolved.

#### 3.4.5 Health and living‐related issues of complex cases

Based on the monthly data base from the national government, we used 23 items to measure health and living‐related issues of persons with complex care needs, which included health issues (6 items), living‐related issues (4 items), economic‐related issues (4 items), family‐related issues (8 items) and other. The staff chose the appropriate items.

### 3.5 Analysis

After confirming the distribution of the gender and age of each person with complex care needs, we analysed the kind of issues overlapping in each household by calculating the proportion of the need for each issue. We also analysed the length of care period required to resolve each primary health issue using survival analysis for each household type. By using survival analysis, especially for the log‐rank test, we replaced survival and death with problem continuation and problem‐solving and analysed the differences in the length of care period needed to resolve the primary health issue in each household. We used SPSS for Windows (version 24; IBM Corp, Armonk, NY, USA) for this analysis with a probability level of less than 0.05 indicating statistical significance.

### 3.6 Ethics

The Institutional Review Board approved this study at the institute of the principal researcher (approval no. NIPH‐IBRA#12260) in 2019. The study was performed in line with the principal of the Declaration of Helsinki. The directors of the centre and the legal department of City A also approved the conduction of this study.

## 4 RESULTS

### 4.1 Characteristics of persons and their households

Table 1 shows the characteristic of persons and their households. Among 155 households, the total number of persons with complex care needs were 227, of which 111 were males (48.9%), and the mean persons' age was 53.9 years (*SD* = 16.8). More than 70% of the cases lived in their own homes at the end of July 2019. The most common type of household was an elderly person with a single child (*N* = 47, 30.3%). Overall, approximately 30% involved persons with disabilities needing support for whom the public health centre/community health centre was the first line of support. More than 70% of the cases' primary health issues had not been resolved by the end of July 2019. Overall, the average length of care period required to resolve their primary health issues was 147.7 (*SD* = 120.6).

**TABLE 1:** Characteristics of persons and their households

	*N* (%)
Persons with complex care needs (*N* = 227)	
Gender	
Male	111 (48.9)
Female	115 (50.7)
Unknown	1 (0.4)
Age	
≤19	4 (1.8)
20s	23 (10.1)
30s	28 (12.3)
40s	32 (14.1)
50s	47 (20.7)
60s	28 (12.3)
70s	31 (13.7)
80s	33 (14.5)
Unknown	1 (0.4)
Mean (*SD*)	53.9 (16.8)
Living places of patients with complex cases at the end of July 2019	
Own home	163 (71.8)
Admission to a nursing home	15 (6.6)
Admission to a hospital	35 (15.4)
Death/Move‐out	13 (5.7)
Unknown	1 (0.4)
Households (*N* = 155)	
Type of households	
A single‐person <65 years old	45 (29.0)
A single‐person ≥65 years old	17 (11.0)
Elderly couple	4 (2.6)
Single‐mother/father	14 (9.0)
Elderly person with a single child	47 (30.3)
A three‐generation	4 (2.6)
Other	24 (15.5)
First consultant departments	
A department of supporting persons with disabilities	54 (34.8)
Public health centre/community health centre	47 (30.3)
A department of elderly care	29 (18.7)
Welfare office	18 (11.6)
Other	7 (4.5)
Whether the primary health issues had not been resolved by the end of July 2019	
Resolved	43 (27.7)
Unresolved	112 (72.3)
The average length of care period required to resolve their primary health issues	
Mean (*SD*)	147.7 (120.6)

### 4.2 The proportion of overlapping health and living‐related issues in each household

Table 2 shows the proportion of overlapping health and living‐related issues in each household. Mental illness and economic distress overlapped as the most common issues. Additionally, neighbourhood trouble, abuse, domestic violence and deteriorating family relationships also overlapped within each household.

**TABLE 2:** The proportion of overlapping health and living‐related issues in each household (*N* = 155)

	A single‐person household							
	Total (*N* = 155)	< 65 years old (*N* = 45)	≥ 65 years old (*N* = 17)	Elderly couples (*N* = 4)	Single‐mother/father (*N* = 14)	Elderly person with a single child (*N* = 47)	A three‐generation (*N* = 4)	Other (*N* = 24)
Health issues								
Illness/Injury	31 (20.0)	1 (2.2)	5 (29.4)	2 (50.0)	1 (7.1)	16 (34.0)	2 (50.0)	4 (16.7)
Physical disability	19 (12.3)	3 (6.7)	2 (11.8)	0 (0.0)	1 (7.1)	10 (21.3)	0 (0.0)	3 (12.5)
Intellectual disability	34 (21.9)	6 (13.3)	0 (0.0)	1 (25.0)	9 (64.3)	10 (21.3)	1 (25.0)	7 (29.2)
Mental illness	112 (72.3)	37 (82.2)	9 (52.9)	3 (75.0)	6 (42.9)	36 (76.6)	2 (50.0)	19 (79.2)
Dementia	19 (12.3)	0 (0.0)	4 (23.5)	1 (25.0)	1 (7.1)	8 (17.0)	1 (25.0)	4 (16.7)
Addiction	17 (11.0)	6 (13.3)	3 (17.6)	0 (0.0)	1 (7.1)	5 (10.6)	0 (0.0)	2 (8.3)
Living‐related issues								
Unsanitary; trash in the house	15 (9.7)	2 (4.4)	1 (5.9)	1 (25.0)	3 (21.4)	7 (14.9)	0 (0.0)	1 (4.2)
Neighbourhood trouble	24 (15.5)	9 (20.0)	5 (29.4)	2 (50.0)	1 (7.1)	7 (14.9)	0 (0.0)	0 (0.0)
Social isolation	3 (1.9)	1 (2.2)	0 (0.0)	1 (25.0)	0 (0.0)	1 (2.1)	0 (0.0)	0 (0.0)
Refusing the support	3 (1.9)	1 (2.2)	0 (0.0)	0 (0.0)	0 (0.0)	1 (2.1)	0 (0.0)	1 (4.2)
Economic‐related issues								
Unemployment	3 (1.9)	2 (4.4)	0 (0.0)	0 (0.0)	1 (7.1)	0 (0.0)	0 (0.0)	0 (0.0)
Unstable work	9 (5.8)	3 (6.7)	0 (0.0)	0 (0.0)	1 (7.1)	3 (6.4)	2 (50.0)	0 (0.0)
Multiple debts	9 (5.8)	2 (4.4)	1 (5.9)	0 (0.0)	0 (0.0)	6 (12.8)	0 (0.0)	0 (0.0)
Economic distress	41 (26.5)	12 (26.7)	8 (47.1)	0 (0.0)	4 (28.6)	10 (21.3)	2 (50.0)	5 (20.8)
Family‐related issues								
Abuse	26 (16.8)	2 (4.4)	0 (0.0)	1 (25.0)	6 (42.9)	10 (21.3)	3 (75.0)	4 (16.7)
Difficulties raising children at home	7 (4.5)	0 (0.0)	0 (0.0)	1 (25.0)	2 (14.3)	1 (2.1)	2 (50.0)	1 (4.2)
School absenteeism	2 (1.3)	0 (0.0)	0 (0.0)	0 (0.0)	1 (7.1)	0 (0.0)	0 (0.0)	1 (4.2)
Withdrawal	15 (9.7)	1 (2.2)	1 (5.9)	1 (25.0)	3 (21.4)	9 (19.1)	0 (0.0)	0 (0.0)
Unemployment of his/her child	16 (10.3)	1 (2.2)	1 (5.9)	0 (0.0)	0 (0.0)	13 (27.7)	1 (25.0)	0 (0.0)
Domestic violence	23 (14.8)	1 (2.2)	1 (5.9)	2 (50.0)	1 (7.1)	12 (25.5)	0 (0.0)	6 (25.0)
Deteriorating family relationships	23 (14.8)	2 (4.4)	0 (0.0)	1 (25.0)	4 (28.6)	11 (23.4)	2 (50.0)	3 (12.5)
Difficulties in home care	18 (11.6)	4 (8.9)	6 (35.3)	0 (0.0)	2 (14.3)	3 (6.4)	1 (25.0)	2 (8.3)
Other	8 (5.2)	1 (2.2)	2 (11.8)	0 (0.0)	1 (7.1)	2 (4.3)	0 (0.0)	2 (8.3)

### 4.3 The length of the care period needed to resolve the primary health issue in each household

Due to the small sample size, we conducted the log‐rank test to analyse whether there was a difference in the length of care period needed to resolve the primary health issues depending on each household: single‐person household <65 years old, ≥65 years old, and elderly person with a single child (Table 3). The results showed that single‐elderly households took less time to solve problems than other households (estimate = 110.2, 95% confidence interval = 48.32–172.01, *p* = .029).

**TABLE 3:** The length of the care period needed to resolve the primary health issue in each household (*N* = 43)

	*N*	Average			*p*
		Estimate	*SE*	95% CI	
A single‐person household <65 years old	11	192.3	21.0	151.18–233.32	.339
Reference = 0	32	159.5	33.0	94.86–224.23	
A single‐person household ≥65 years old	37	110.2	31.6	48.32–172.01	.029**
Reference = 0	6	195.8	19.3	158.00–233.68	
Elderly person with a singlehood child	33	220.7	46.2	130.07–311.33	.151
Reference = 0	10	172.7	18.3	136.83–208.62	
Total	43	183.9	17.7	149.24–218.53	—

Note

Log‐rank test.

Abbreviation: 95% CI, 95% confidence interval.

** *p* < .05 Log‐rank test.

## 5 DISCUSSION

In this cross‐sectional study, we focused on the characteristics of persons with complex care needs in different households in Japan. We found that 30.3% of households were made up of an elderly person with a single child, with mental disorder and economic distress overlapping as the primary health issues. Additionally, it took less time to resolve the primary health issues of single‐elderly persons who lived alone than for elders with single‐child households or single‐person <65‐year‐old households. Parents in their 80s are often economically supporting their single children in their 50s with a mental disorder, non‐attendance at school, and difficulty finding employment that began in their teens and 20s, which was prolonged and unresolved (Cabinet Office, 2019; Ministry of Health, Labour, & Welfare, 2019). With the increasing care needs of parents, the poverty of children whom they are required to support has become visible. This situation is named the ‘‘8050 issue” and has been gradually increasing in Japan with households of elderly persons with a single child being subjected to social isolation. Thus, to prevent the likelihood of social isolation, existing health systems and policies mainly targeted single‐person households with residents 65 and older as well as elderly couple households (Yoshioka‐Maeda, 2020). Thus, elders with single‐child households are uncovered by existing health systems and policies based on the assumption that adult children care for them.

Additionally, mental illness was one of the factors of persons’ health complexity (Loeb et al., 2016; Mount et al., 2015). Furthermore, poverty was a related factor of mortality and degrees of deprivation were deeply linked to low life‐control and reduced opportunities for social participation (Marmot, 2017; Sylvestre et al., 2018). These issues were one of the social determinants of health (Marmot, 2017; Singer et al., 2019). Our finding suggested that elders with single‐child households overlapped with hidden issues of poverty, mental disorders and social isolation in the community. Local government should thus enrich holistic healthcare systems and policies for elders with single‐child households in each community to prevent social isolation, provide early detection for their disability and improve the social gradient. Also, PHNs should assess their persons’ mental health condition and economic status at the initial stage of care considering these issues are often interlinked with persons' health conditions.

We also found that health and living‐related issues, such as abuse, domestic violence, family dysfunction, and neighbourhood trouble, also overlapped in each household with more than 70% of persons not resolving their primary health issue. Previous studies mainly focused on sociodemographic status and multimorbidity of persons who had high medical and social care needs (Baker et al., 2018; Bunn et al., 2018; Palladino et al., 2016). Furthermore, family dysfunction was a common factor of domestic violence and abuse (Chan et al., 2019; Kageyama et al., 2018; Lino et al., 2019). Despite these issues being thought of as taboo (Simone et al., 2016), a multidisciplinary team approach, social capital factors and community participation were mediating factors for domestic violence and abuse (Cao & Maguire‐Jack, 2016; Isumi et al., 2018; Koga et al., 2020; Lucero et al., 2019; Pillemer et al., 2016). Additionally, a previous study showed that community‐based approaches enhanced social cohesion in the neighbourhood (Shen et al., 2017). Our results suggested that health policies and PHNs should focus on the crucial effect of the community context on health and in strengthening the social cohesion of each household with complex care needs by using a grassroots movement approach to resolve linked health issues.

### 5.1 Limitations

There were several limitations in this study. Firstly, the study sample was small and limited, being from only one municipality in Japan. Due to limited resources, we could only collect data from April 2018–the end of July 2019. Additionally, we knew that the number of cases of the centre was small when designing this collaborative research study; thus, we could not calculate the sample size. This is the main reason that we could not conduct the log‐rank test to analyse whether there was a difference in the length of care period needed to resolve the primary health issue in all households. Therefore, our results may be limited in terms of generalizing the results to other municipalities in Japan.

Secondly, we used registered data that were reported to the national government. We used a national data format for collecting data; therefore, we could not validate this instrument. Furthermore, this format did not include the educational background of each case and what kind of support was provided by the centre. As the support provided by the centre, depended on the tacit knowledge of staff, this knowledge regarding the kind of support that should be provided to support households of persons with complex care needs should be clarified. Additionally, in the future, a cohort study should be conducted to identify the cause–effect relationship between complex cases and their health issues.

## 6 CONCLUSION

The findings of this study suggested that elders with single‐child households overlapped with hidden issues of poverty, mental disorder and social isolation in the community and were uncovered by existing healthcare systems. Furthermore, mental illness and economic distress overlapped as the most common issues faced by these households. Overall, these findings have highlighted the importance of understanding the characteristics of households of persons with complex care needs. Therefore, to improve the social gradient of persons with complex care needs, national and local governments should use these findings to guide the development of holistic healthcare systems and policies, especially for elderly‐with‐single‐child households, in each community. Additionally, to reduce health inequities and resolve interlinked complex health issues of each household, PHNs play a crucial role in local government in developing holistic community‐based approaches and needs‐oriented health policies.

## CONFLICT OF INTEREST

The authors declare that they have no competing interests.

## AUTHOR CONTRIBUTION

All authors listed meet the authorship criteria according to the guidelines of the International Committee of Medical Journal Editors. KYM and HF: Study design, collection and management of the data. HF: Statistical data analysis. KYM and HF: Interpretation of results. KYM: Manuscript drafting, and KYM and HF: Manuscript revision for important intellectual content. All authors approved their manuscript of final submitted version.

## Data Availability

The data of this study are not publicly available due to strictly protect the privacy of research participants.
