# Corrigendum

**DOI:** 10.1002/nop2.1210

**Published:** 2022-06-13

In the article by Yoshioka‐Maeda and Fujii (2020), the author found mistakes in Table 3, which are corrected in this version.

**TABLE 3:** Length of the care period needed to resolve the primary health issue in each household (*N* = 43)

	Average					
	*n*	Estimate	SE	95% CI	*p*	
A single‐person household <65 years old	11	159.5	33.0	(94.86–224.23)	.339	
Reference = 0	32	192.3	21.0	(151.18–233.32)		
A single‐person household ≥65 years old	6	110.2	31.6	(48.32–172.01)		.029**
Reference = 0	37	195.8	19.3	(158.00–233.68)		
Elderly person with a singlehood child	10	220.7	46.2	(130.07–11.33)		.151
Reference = 0	33	172.7	18.3	(136.83–208.62)		
Total	43	183.9	17.7	(149.24–218.53)		–

Note

Log‐rank test.

95%CI, 95% confidence interval.

** *p* < .05 Log‐rank test.

In Table 3, the data under the headings Estimate, SE and 95% CI in rows one and two were reversed. The data under the heading n were reversed in rows three and four and in rows five and six. The corrected Table 3 appears below.

We apologize for these errors.
